# Editorial: Advanced Deep-Transfer-Leveraged Studies on Brain-Computer Interfacing

**DOI:** 10.3389/fnins.2021.733732

**Published:** 2021-08-19

**Authors:** Yizhang Jiang, Yu-Dong Zhang, Mohammad Khosravi

**Affiliations:** ^1^School of Artificial Intelligence and Computer Science, Jiangnan University, Wuxi, China; ^2^Department of Informatics, University of Leicester, Leicester, United Kingdom; ^3^Department of Computer Engineering, Persian Gulf University, Bandar Bushehr, Iran

**Keywords:** deep learning, transfer learning, brain-computer interfacing, EEG, neuroscience

Brain-computer interfacing (BCI) has attracted rapidly increasing research interest in the last decade due to recent advances in neurosciences, wearable/mobile biosensors, and analytics. The ultimate goal is to provide a pathway from the brain to the external world via mapping, assisting, augmenting, or repairing human cognitive or sensory-motor functions. Recently, many advanced machine learning technologies have appeared, such as deep learning, transfer learning, and so on. The deep learning method has achieved great success in the image and the video analysis, the natural language processing, the speech recognition, etc., and recently has also started to find applications in BCI. Transfer learning, which improves learning in a new task by leveraging data or knowledge from other relevant tasks, can be particularly useful in BCI to cope with variability across individuals or tasks, accelerating learning and improving performance. Advanced deep-transfer-leveraged learning technologies can also be integrated to take advantage of both domains.

Although the studies of BCI using advanced deep learning and/or transfer learning methods become more and more popular, there still exist many unsolved fundamental problems so far, such as deep learning representation of some EEG-based BCI data from multiple modalities, mapping data from one modality to another to achieve cross-source BCI data analysis, identifying, and utilizing relations between elements from two or more different signal sources for comprehensive BCI data analysis, fusing information from two or more signal sources to perform a more accurate prediction, transferring knowledge between modalities and their representations, and recovering missing modality data given the observed ones.

In the past decade, several EEG-based BCI methods and technologies have been developed and shown promising results in some real-world examples such as neuroscience, medicine, and rehabilitation, which led to a proliferation of papers showing accuracy/performance and comparison, but most do not advance to real-time, translation, or application. Then, these papers do not fare well, either because of lack of novelty (known technique) or no bio/med/experiment/clinical translation. For all the reasons mentioned above, it inspires us to exploit and develop effective advanced deep learning and/or transfer learning algorithms for addressing fundamental issues in BCI and rehabilitation fields.

This Research Topic (RT) of the Frontiers in Neuroscience (section: Neuroprosthetics) is a selection of 22 papers presented at the RT “Advanced Deep-Transfer-Leveraged Studies on Brain-Computer Interfacing.” We provide a brief summary of these papers as follows.

## BCI Approaches

The brain computer interface (BCI) is a direct connection between human or animal brain (and brain cell culture) and external devices. In this RT, some scholars proposed many novel methods on BCI technologies. For example, Huang et al. proposed a classification method using sparse representation (SR) and fast compression residual convolutional neural networks (FCRes-CNNs). They obtained the features of the EEG signal through the common spatial patterns algorithm, and they constructed the redundant dictionary with sparse representation based on these features. Subsequently, they used sparse features as the input of fast compression deep learning network to classify EEG signals. Meanwhile, Zhang W. et al. proposed an EEG phase-dependent closed-loop mechanical vibration stimulation method. This novel method is an improvement on traditional vibration stimulation enhancement research and helps to make the stimulation more precise and effective.

BCI has been regarded as a newly developing intervention in promoting motor recovery in stroke survivors. Several studies have been performed in chronic stroke to explore its clinical and subclinical efficacy. However, evidence in subacute stroke was poor, and the longitudinal sensorimotor rhythm changes in subacute stroke after BCI with exoskeleton feedback were still unclear. In this regard, Chen S. et al. studied the longitudinal sensorimotor rhythm changes of BCI with exoskeleton feedback in subacute stroke. The experiments showed that The BCI group showed larger percentage points of improvement and good motor recovery than the control group.

In theory, the BCI system can monitor the signals generated by neural activities through a variety of sensors and other signal acquisition equipment. Through the analysis and processing of the signals, the signals are classified according to separate thinking activities to generate corresponding control commands to complete the interactive tasks between users and external devices. To achieve this goal, Wang et al. explored the classification method of EEG signals based on a multilayered neural network that plays an important role in promoting the practicality of the BCI. In this article, they developed the left-handed and right-handed motor imagination EEG classification model based on convolution neural network, and they achieved 75.3% classification accuracy on the test set of BCI common data set. The designed model can be transplanted to mobile phones, computers, tablets, and other terminal devices, used in BCI technology, medical rehabilitation, the field of healthcare. Meanwhile, Chen Y. et al. developed a knowledge-leverage-based support matrix machine (KL-SMM) to improve the classification performance when only a few labeled EEG data in the target domain (target subject) were available. Different from most current model parameter transfer learning methods, the proposed method can propagate the structural information from the source model to the target model. In addition, the proposed method can afford privacy protection by leveraging only the model knowledge of the source domain.

## Eeg Approaches

Electroencephalogram (EEG) as a biomarker plays an important role in the BCI. The EEG signal can be used as a basis for the prediction of brain behavior and diagnoses of disease. For example, EEG signals are often used to determine the presence and type of epilepsy in clinical diagnosis. In this Research Topic, several papers focus on using different advanced artificial intelligence methods to identify epileptic seizures via EEG signal. Zhou and Li analyzed EEG signal features from linear and non-linear perspectives, and dynamically extract effective features using an improved radial basis function neural network. Moreover, they introduced one against one strategy classifier to reduce the probability of error classification.

Because the responses to EEG signals of different patients in the same cognitive activity show a certain degree of similarity, Zhang Y. et al. leveraged abundant labeled EEG epochs from a related source domain and reused them in the target domain. They proposed an online selective transfer TSK fuzzy classifier underlying joint distribution adaptation and manifold regularization. Their classifier can make use of very few calibration data in the target domain to induce the target predictive function. Using joint distribution adaptation to minimize the marginal distribution distance and conditional distribution distance between the source and target domains, the computational complexity of the classifier can be reduced. Meanwhile, Zhang G. et al. proposed a multi-scale non-local (MNL) network to achieve automatic EEG signal detection. Their MNL-Network is based on 1D convolution neural network involving two specific layers to improve the classification performance. One layer is named the signal pooling layer and the other layer is called a multi-scale non-local layer. The experimental results demonstrate that the MNL-Network could achieve competitive results in the EEG classification task. Additionally, Jiang K. et al. studied how to select effective EEG features to guarantee high-efficiency artificial intelligence-assisted clinical diagnosis. They constructed a stacked deep structure for feature selection in a layer-by-layer manner so as to add random projections into the original features, so that the manifold structure existing in the original feature space was continuously opened in a stacked way. Therefore, according to the stacked generalized principle, the original input feature space became more linearly separable. Moreover, Ni et al. proposed a noise-insensitive Takagi-Sugeno-Kang (TSK) fuzzy system for EEG signal recognition. In particular, they developed a possibilistic clustering in Bayesian framework with interclass competitive learning to determine antecedent parameters of fuzzy rules. To further promote the noise insensitivity of rules, they used the asymmetric expectile term and Ho-Kashyap procedure to learn the consequent parameters of rules. Comprehensive experiments on Bonn EEG dataset revealed that the proposed fuzzy system achieved robust and effective performances for EEG signal recognition. Besides, Xu et al. proposed a one-dimensional convolutional neural network long and short-term memory (1D CNN-LSTM) model to automatically recognizes epileptic seizures through EEG signal analysis. In short, a one-dimensional convolutional neural network (CNN) and long and short-term memory model are used to extract temporal features from standardized EEG sequence data. Several fully connected layers are used for epilepsy recognition.

To exploit the diversity and complementariness of different feature representations of EEG signals, Xue et al. developed a new auto-weighted multi-view discriminative metric learning method with Fisher discriminative and global structure constraints. On the one hand, they exploited the multiple features of different views in the scheme of the multi-view feature representation. On the other hand, they considered both the Fisher discriminative constraint and global structure constraint into the discriminative metric space, in which the intraclass EEG signals were compact, and the interclass EEG signals were separable as much as possible. Meanwhile, Dong et al. developed a method combining the non-negative matrix factorization technology and transfer learning. They reported that the non-negative matrix factorization can assure to obtain essential information between the testing and the training dataset, and the combination of shared subspace and the original feature space can fully use of the testing signals and the training signals.

Accurate and automatic classification of the speech imagery EEG signals from the BCI system is highly demanded in clinical diagnosis. The key factor in designing an automatic classification system is to extract essential features from the original input. To achieve this goal, Zhang, Luo et al. proposed a dynamic multi-scale network. The whole classification network was based on ResNet, and the input signal first encoded the features by the Short-time Fourier Transform (STFT). Finally, they incorporated a dynamic multi-scale layer to allow the network to learn multi-scale features from different receptive fields at a more granular level.

Currently, the recognition method of EEG signals is one of the important technology for human emotion recognition. The traditional machine learning method has a major disadvantage in that the feature extraction process is usually cumbersome. Zhang Y. et al. focus on emotion recognition based on EEG signals using deep learning model combination. They studied the application of several deep learning models in the research field of EEG-based emotion recognition, including deep neural networks (DNN), convolutional neural networks (CNN), long short-term memory (LSTM), and a hybrid model of CNN and LSTM (CNN-LSTM). Then they used four deep learning models to learn and predict emotion recognition on the DEAP EEG dataset.

Changes in physiological functions during sleep lead to corresponding changes in EEG signals. The acquisition and processing of patients' sleep data at night need the help of automation and digital technology. Wen proposed a sleep quality detection method based on EEG signals. He used the discrete wavelet transform (DWT) for feature extraction and adopted the transfer learning support vector machine (TL-SVM) for classifying the feature data. The proposed method was tested using 60 pieces of data from the National Sleep Research Resource Library of the United States, and the experimental results show that the classification performance of the TL-SVM classifier is significantly higher than other comparison algorithms.

## Clinic Applications

In clinic medicine files, the computer-aided diagnosis of brain diseases technology began to use some existing advanced machine learning methods. For example, Cai et al. studied altered patterns of functional connectivity and causal connectivity in salience subnetwork of subjective cognitive decline and amnestic mild cognitive impairment. Meanwhile, Jiang C. et al. studied the numerical evaluation of the influence of skull heterogeneity on transcranial ultrasonic focusing. They investigated how the focus deviates after phase-aberration compensation with ray tracing using time-reversal theory. They simulated the propagation of ultrasound for transcranial focusing with the k-space pseudo spectral method. The results revealed minimal deviation in the focal region and suggested that transcranial focusing deflections are caused mostly by ultrasonic refraction on the surface of the skull bone. Besides, Cai et al. analyzed the altered patterns of phase position connectivity in default mode subnetwork of subjective cognitive decline and amnestic mild cognitive impairment. Vestibular migraine (VM) is a multidisciplinary disease under exploration. Moreover, Yan et al. investigated the clinical features of VM under three temporal patterns. They found that vestibular stimulation could inhibit the trigeminal pain pathway, while painful trigeminal stimulation could excite the vestibular system. This finding may contribute to the clinical identification of VM and further clarification of its pathogenesis. Furthermore, Hong et al. proposed a feature fusion and attention network (FFA-DMRI) is proposed to separate noise from brain magnetic resonance imaging (MRI). Inspired by the attention-guided convolutional neural networks (CNN) network and convolutional block attention module, they designed a spatial attention mechanism to obtain the area of interest in MRI. Furthermore, they marked full use of the multilevel structure and boost the expressive ability of network by the feature fusion block. The comprehensive experiments on Alzheimer's disease neuroimaging initiative dataset demonstrated high effectiveness of FFA-DMRI with maintaining the crucial brain details. Additionally, Ji et al. developed a method to construct dynamic brain functional networks (DBFNs) via hyper-graph manifold regularization (HMR), and employed it to classify mild cognitive impairment (MCI) subjects. Finally, they conduct classification experiments to classify MCI subjects from normal subjects to verify the effectiveness of our method.

## Conclusion

This Research Topic focuses primarily on novel theories and methods proposed for EEG single and health information processes. New methods can be used to diagnose brain neurological diseases predict and inhibit the onset of epilepsy, and can also be used for some clinic applications. As can be appreciated, these articles covered a wide range of advances and new insights in our understanding of the brain neuroscience.

## Author Contributions

All authors listed have made a substantial, direct and intellectual contribution to the work, and approved it for publication.

## Conflict of Interest

The authors declare that the research was conducted in the absence of any commercial or financial relationships that could be construed as a potential conflict of interest.

## Publisher's Note

All claims expressed in this article are solely those of the authors and do not necessarily represent those of their affiliated organizations, or those of the publisher, the editors and the reviewers. Any product that may be evaluated in this article, or claim that may be made by its manufacturer, is not guaranteed or endorsed by the publisher.

